# Computed Tomography-Based Radiomics in Predicting T Stage and Length of Esophageal Squamous Cell Carcinoma

**DOI:** 10.3389/fonc.2021.722961

**Published:** 2021-10-14

**Authors:** Mingwei Yang, Panpan Hu, Minglun Li, Rui Ding, Yichun Wang, Shuhao Pan, Mei Kang, Weihao Kong, Dandan Du, Fan Wang

**Affiliations:** ^1^ Department of Radiation Oncology, The First Affiliated Hospital of Anhui Medical University, Hefei, China; ^2^ Department of Radiotherapy, The First Affiliated Hospital of University of Science and Technology of China (USTC), Division of Life Sciences and Medicine, University of Science and Technology of China, Hefei, China; ^3^ Department of Radiation Oncology, University Hospital, Ludwig-Maximilians-University (LMU) Munich, Munich, Germany; ^4^ Department of Occupational and Environmental Health, School of Public Health, Anhui Medical University, Hefei, China; ^5^ Department of Emergency Surgery, Department of Emergency Medicine, The First Affiliated Hospital of Anhui Medical University, Hefei, China; ^6^ Department of Radiology, The First Affiliated Hospital of Anhui Medical University, Hefei, China

**Keywords:** esophageal squamous cell carcinoma, radiomic, CT, tumor T stage, tumor length

## Abstract

**Background:**

Because of the superficial and infiltrative spreading patterns of esophageal squamous cell carcinoma (ESCC), an accurate assessment of tumor extent is challenging using imaging-based clinical staging. Radiomics features extracted from pretreatment computed tomography (CT) or magnetic resonance imaging have shown promise in identifying tumor characteristics. Accurate staging is essential for planning cancer treatment, especially for deciding whether to offer surgery or radiotherapy (chemotherapy) in patients with locally advanced ESCC. Thus, this study aimed to evaluate the predictive potential of contrast-enhanced CT-based radiomics as a non-invasive approach for estimating pathological tumor extent in ESCC patients.

**Methods:**

Patients who underwent esophagectomy between October 2011 and September 2017 were retrospectively studied and included 116 patients with pathologically confirmed ESCC. Contrast-enhanced CT from the neck to the abdomen was performed in all patients during the 2 weeks before the operation. Radiomics features were extracted from segmentations, which were contoured by radiologists. Cluster analysis was performed to obtain clusters with similar radiomics characteristics, and chi-squared tests were used to assess differences in clinicopathological features and survival among clusters. Furthermore, a least absolute shrinkage and selection operator was performed to select radiomics features and construct a radiomics model. Receiver operating characteristic analysis was used to evaluate the predictive ability of the radiomics signatures.

**Results:**

All 116 ESCC patients were divided into two groups according to the cluster analysis. The chi-squared test showed that cluster-based radiomics features were significantly correlated with T stage (*p* = 0.0254) and tumor length (*p* = 0.0002). Furthermore, CT radiomics signatures exhibited favorable predictive performance for T stage (area under the curve [AUC] = 0.86, sensitivity = 0.77, and specificity = 0.87) and tumor length (AUC = 0.95, sensitivity = 0.92, and specificity = 0.91).

**Conclusions:**

CT contrast radiomics is a simple and non-invasive method that shows promise for predicting pathological T stage and tumor length preoperatively in ESCC patients and may aid in the accurate assessments of patients in combination with the existing examinations.

## Introduction

Esophageal cancer is the eighth most common cancer and the sixth leading cause of cancer-related death worldwide, which presents a major global health challenge (1, 2). Esophageal squamous cell carcinoma (ESCC) accounts for more than 90% of esophageal malignancies in China and has a poor prognosis and an unsatisfactory survival rate (3). Multidisciplinary treatment strategies are recommended for ESCC patients to improve therapeutic outcomes, especially for those in advanced tumor stages. Because of the crucial role of accurate staging for guiding treatment decisions, a sophisticated staging approach, which includes endoscopy, endoscopic ultrasound (EU), and contrast-enhanced computed tomography (CT), is now in routine use. However, staging accuracy remains extremely low, even with the help of modern positron emission tomography (PET) imaging (4, 5). Especially for primary tumors, an accurate determination of tumor depth and length can be extremely difficult in some cases, primarily due to the superficial and infiltrative spreading pattern of ESCCs. Thus, there is an urgent need for the development of a novel approach for improved staging accuracy.

Radiomics is a non-invasive method to convert imaging data into high-resolution quantitative features using automatic algorithms (6). Currently, CT-based radiomics features, including texture, wavelet, and fractal features, have shown promise for predicting multiple tumors (7, 8). Several studies on the potential role of radiomics in ESCC have been conducted; however, most have focused on treatment response (9), and its ability for accurate tumor staging has not yet been comprehensively explored in patients with ESCC. Therefore, we aimed to examine the predictive potential of radiomics features and develop radiomics signatures to evaluate clinicopathological features and prognosis of ESCC patients preoperatively, which will offer a non-invasive, simple, and economical approach for the pretreatment assessment of ESCC in clinical practice.

## Materials and Methods

### Patient Selection

**Figure 1:**
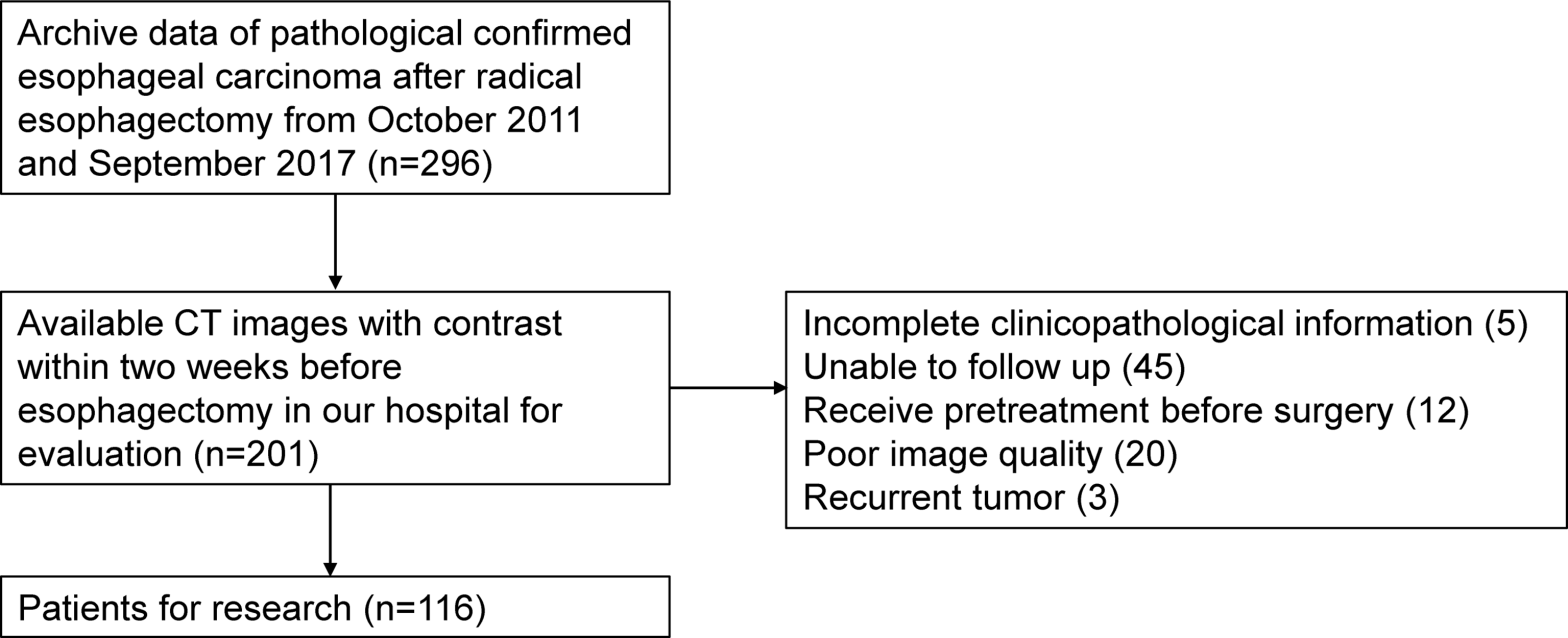
Selection flow of ESCC patients.

Patients who underwent esophagectomy between October 2011 and September 2017 at the First Affiliated Hospital of Anhui Medical University were retrospectively studied. Inclusion criteria were as follows: 1) underwent radical esophagectomy and ESCC confirmed pathologically, 2) underwent contrast-enhanced CT examination from neck to abdomen during the 2 weeks before esophagectomy, 3) complete clinicopathological information was available, and 4) treatment outcome could be followed up. Exclusion criteria were as follows: 1) received previous treatment before surgery, 2) recurrent tumor, and 3) poor quality of images. A total of 116 patients were enrolled, and the selection flow is presented in **Figure 1**. The study was approved by the local ethics committee, and the need to obtain informed consent was waived.

### Clinicopathological Characteristics Collection and Follow-up

Clinical characteristics of each patient were obtained from the electronic medical system. Histological results were reviewed to gather pathological tumor feature information, which included T stage, N stage, length, histological grade (HG), vascular invasion (VI), and perineural invasion. Each patient was followed up every 3 months for the first year, every 4–6 months for the second year, then annually thereafter. Overall survival (OS) was determined as the time from surgery to death, and progression-free survival (PFS) was determined as the time from surgery to the first progression.

### Image Acquisition and Segmentation

CT scans from neck to abdomen were performed using a 64-channel multi-detector CT scanner (LightSpeed VCT, GE Medical Systems, Milwaukee, WI, USA) before and 30 s after intravenous injection of 80 ml of iodine contrast agent (Ultravist 370; Bayer Schering Pharma, Berlin, Germany). The following acquisition parameters were used for each CT scan: slice thickness 5 mm, reconstruction interval 5 mm, tube voltage 120 kVp, tube current 80–300 mA, high-resolution matrix size 512 × 512, field of view 500 mm, rotation time 0.75 s, pitch 0.938, and pixel size 1.46 mm.

**Figure 2:**
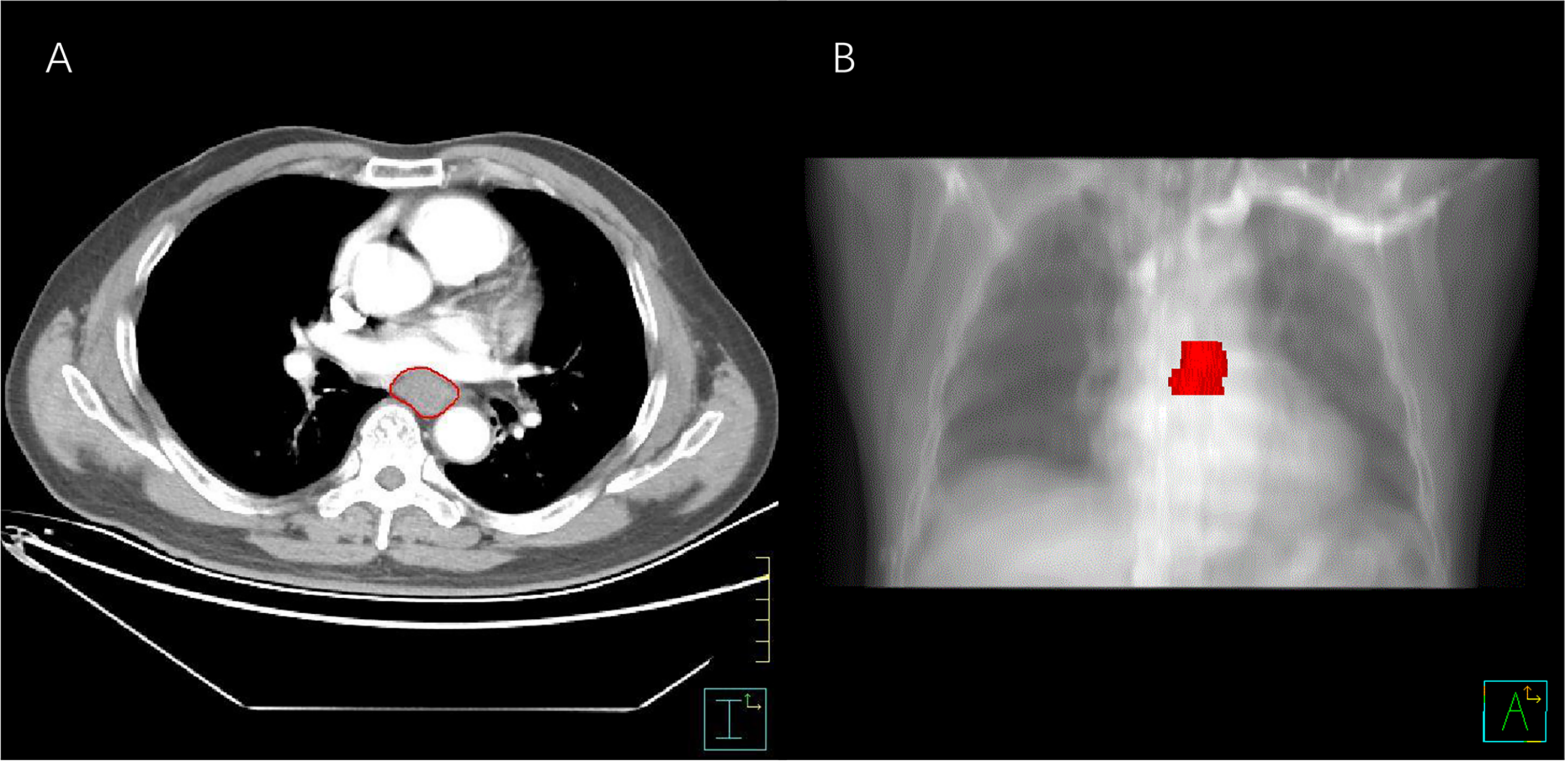
The process of segmentation. **(A)** VOI contouring on an enhanced axial CT slice. **(B)** Three-dimensional image of esophageal cancer. VOI, volume of interest.

**Figure 3:**
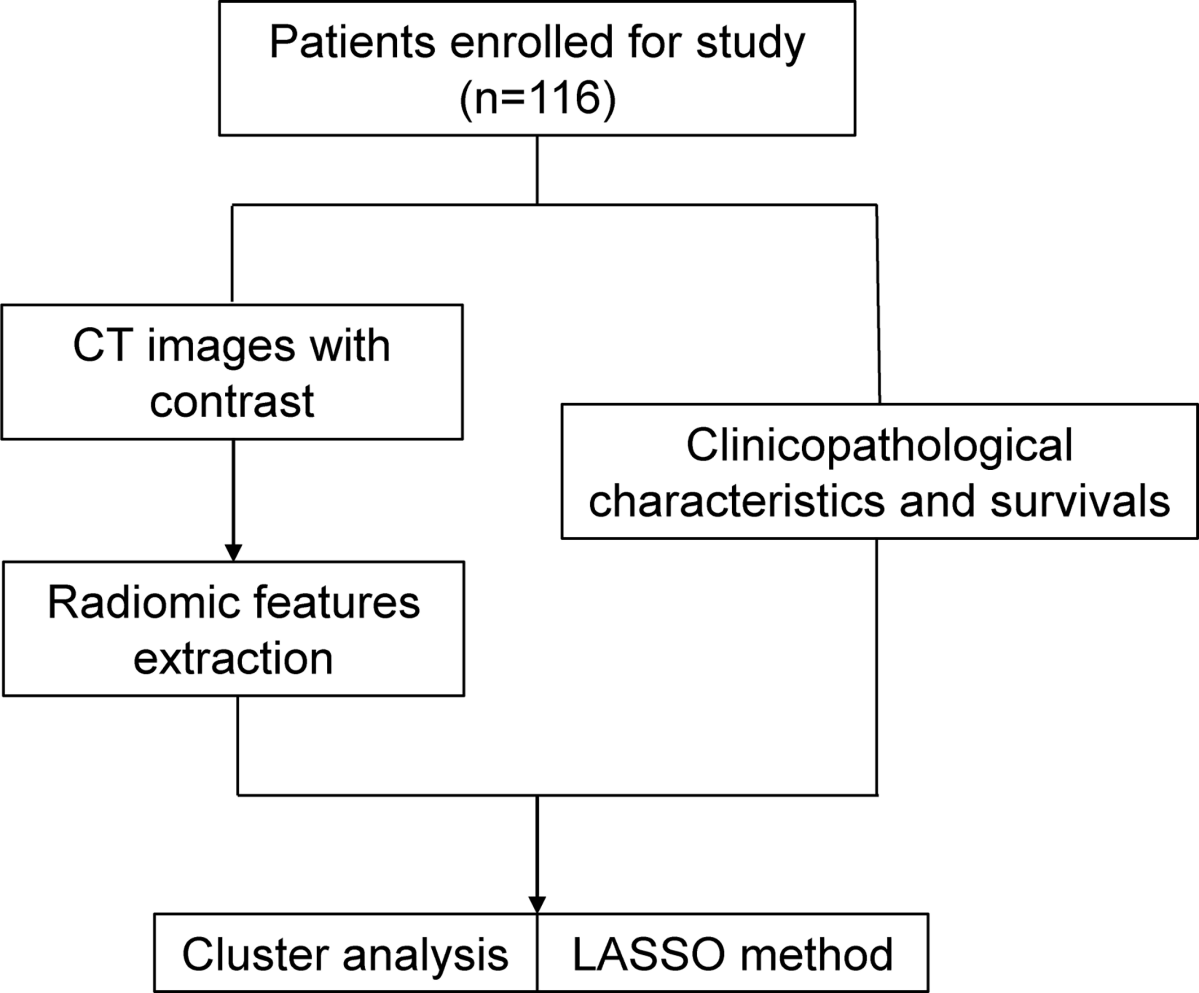
Radiomics analysis workflow.

Contrast-enhanced CT images of each patient were imported from the picture archiving and communication system (Carestream, Canada) into the Pinnacle system for contouring the volume of interest (VOI) (10). The VOI was delineated carefully on each slice following the contours of the gross tumor volume by an experienced radiation oncologist (MY) with 7 years’ experience of esophageal carcinoma contouring and examined by another radiation oncologist (FW) with 15 years’ experience of esophageal carcinoma contouring. The air inside the esophagus was excluded (**Figure 2**). Each disagreement was resolved through discussions. The workflow is presented in **Figure 3**.

### Radiomic Feature Extraction

When tumor segmentation was complete, Digital Imaging and Communications in Medicine images with delineations were exported to MatLab R2015a (MathWorks, Natick, MA, USA), and an algorithm was applied to extract the radiomic features from the VOI of each ESCC patient. In total, 251 radiomics features (details of which are listed in **Supplementary Table 1**) were divided into the following groups: 1) gray-level co-occurrence matrix, 2) gray-level run-length matrix, 3) wavelet gray-level co-occurrence matrix, 4) wavelet gray-level run-length matrix, 5) histogram, 6) geometry, and 7) fractal.

### Statistics

All statistical analysis and modeling were performed in R software (version 3.4.0, R Foundation for Statistical Computing, Vienna, Austria). Statistical tests were two-sided and *p* < 0.05 was considered statistically significant.

Following radiomics feature extraction, cluster analysis based on non-negative matrix factorization was performed to assign patients with similar radiomics characteristics to the same group (cluster). Using this unsupervised machine learning method, 116 patients were assigned into two clusters. The differences in clinicopathological characteristics between the two clusters were then tested using chi-square tests.

Further radiomics feature selection and radiomics model building were performed using the least absolute shrinkage and selection operator (LASSO) method, which is a logistic regression method with 10-fold cross-validation to improve prediction accuracy and interpretability. For the LASSO training and validation cohorts, we used 75% and 25% of all patients, respectively. The optimal lambda was determined according to the training cohort, and the leave-one-out cross-validation method was used for selection. The final radiomics model was determined according to the validation cohort. The area under the curve (AUC), sensitivity, and specificity of the radiomics signatures were calculated to evaluate the predictive performance of the radiomics model. Then, using the selected radiomics features, we constructed a nomogram to describe the prediction potential.

## Results

### Patient Details

A total of 116 patients were included in the study, of which 95 patients were male, and 21 were female. Age ranged from 45 to 82 years; 63 patients were aged ≤ 65 years, and 53 patients were aged > 65 years. Patients with a tumor at the upper-middle, middle, middle-lower, and lower sites of the esophagus were 11, 47, 40, and 18, respectively. Each patient’s pathological assessment was reviewed, and tumors were staged according to the eighth edition of the tumor node metastasis (TNM) staging classification for carcinoma of the esophagus and esophagogastric junction of the American Joint Committee on Cancer (11). Two patients were at TNM stage I, 54 patients were at stage II, 49 patients were at stage III, and 11 patients were at stage IV. Details of tumors including T stage, N stage, tumor grade, and length are provided in **Table 1**.

**Table 1:** General clinicopathological characteristics of enrolled patients.

Characteristics	N
Number of patients	116
Sex	
Male	95 (81.9%)
Female	21 (18.1%)
Age (years)	
≤65	63 (54.3%)
>65	53 (45.7%)
Tumor location	
Upper-middle	11 (9.5%)
Middle	47 (40.5%)
Middle-lower	40 (34.5%)
Lower	18 (15.5%)
Tumor grade	
Low	81 (69.8%)
High	35 (30.2%)
T stage	
T1–T2	22 (19.0%)
T3–T4	94 (81.0%)
N stage	
N0–1	91 (78.4%)
N2–3	25 (21.6%)
TNM stage (8th edition, 2017)	
I–II	56 (48.3%)
III–IV	60 (51.7%)
Tumor length (cm)
≤4.5	75 (64.7%)
>4.5	41 (35.3%)

### Cluster Analysis

**Figure 4:**
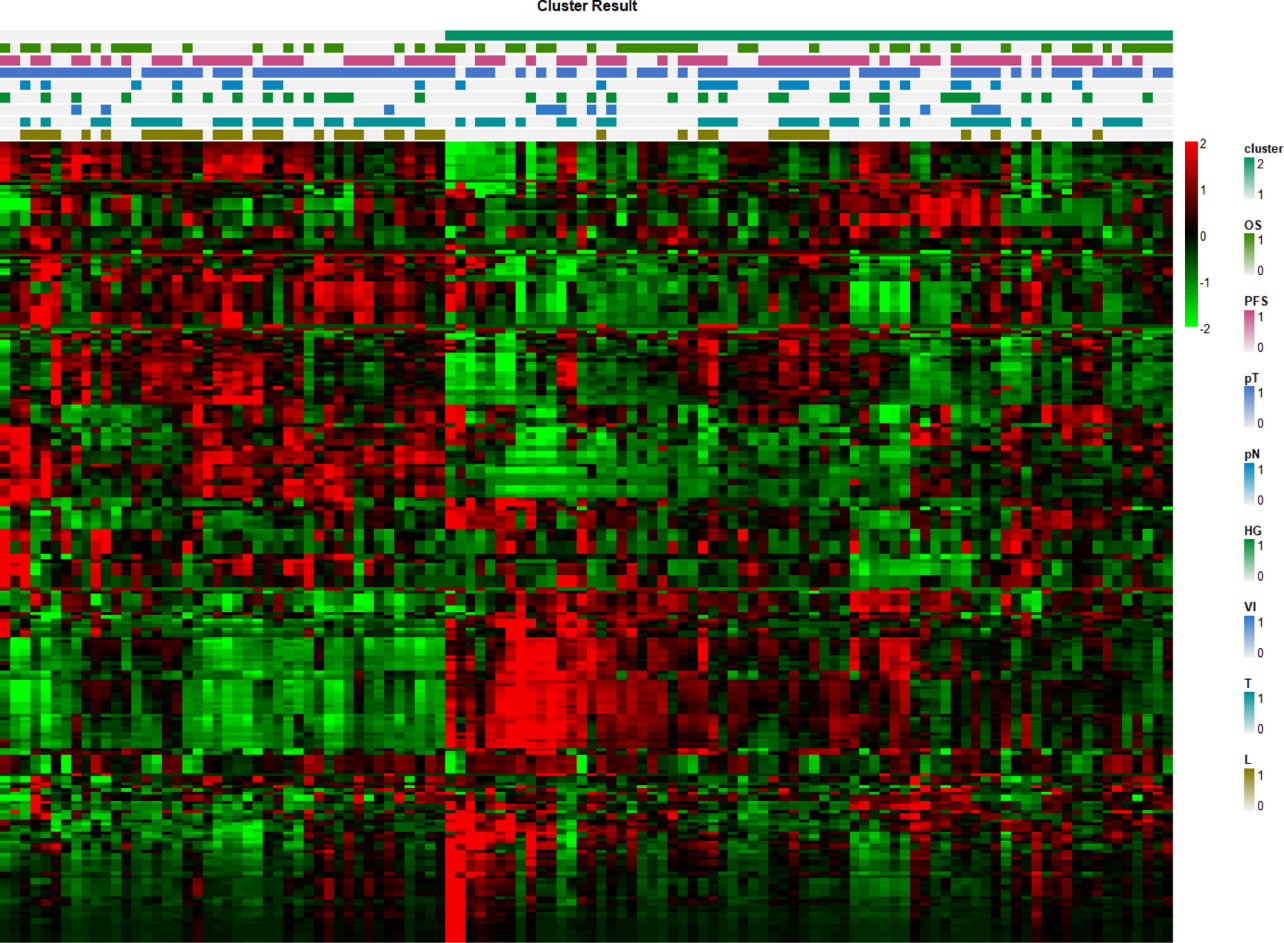
Radiomics feature heatmap. The x-axis represents each patient, and the y-axis represents each radiomics feature.

A total of 116 patients were separated into two clusters by nonnegative matrix factorization according to their radiomics features. As shown in the heatmap in **Figure 4**, patients in the same cluster possessed similar radiomics features. Further chi-square analyses showed that T stage (*p* = 0.0254) and length (*p* = 0.0002) were significantly correlated with the clusters. However, no significant differences were detected for OS, PFS, HG, N stage, tumor stage, or VI between the two clusters (**Table 2**).

**Table 2:** Chi-square analysis of the clinicopathological characteristics between clusters.

Characteristics	χ^2^	p
OS	0.135	0.7134
PFS	0.024	0.8767
pT	4.993	0.0254
pN	0.067	0.7955
HG	0.244	0.6217
VI	2.926	0.0872
TNM stage	0.224	0.6360
Tumor length	14.262	0.0002

OS, overall survival; PFS, progression-free survival; pT, pathological T stage, pN, pathological N stage, HG, histological grade, VI, vascular invasion.

### LASSO Analysis and Nomogram Construction

**Figure 5:**
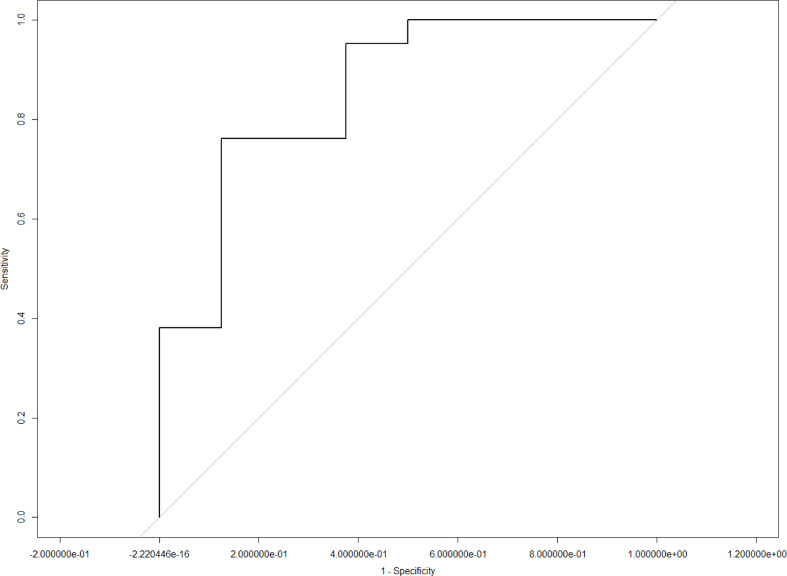
Receiver operating curve analysis for T stage using the radiomics model.

**Figure 6:**
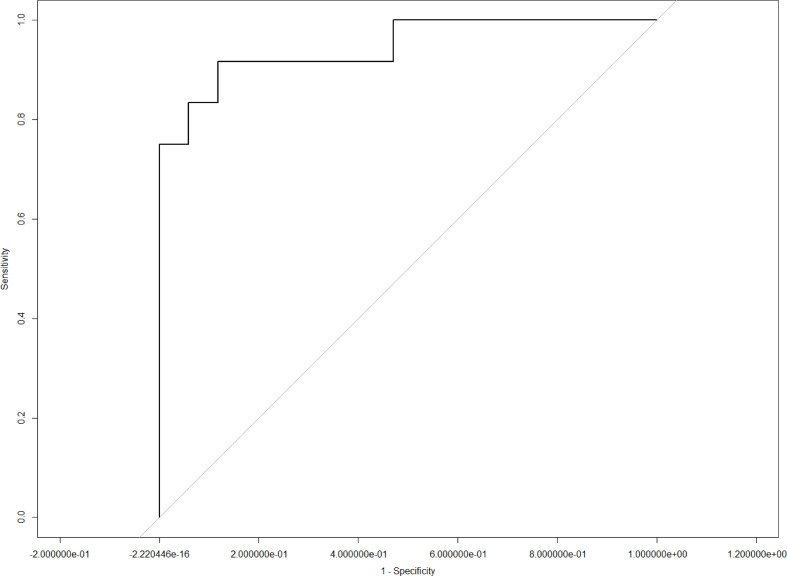
Receiver operating curve analysis for length using the radiomics model.

**Figure 7:**
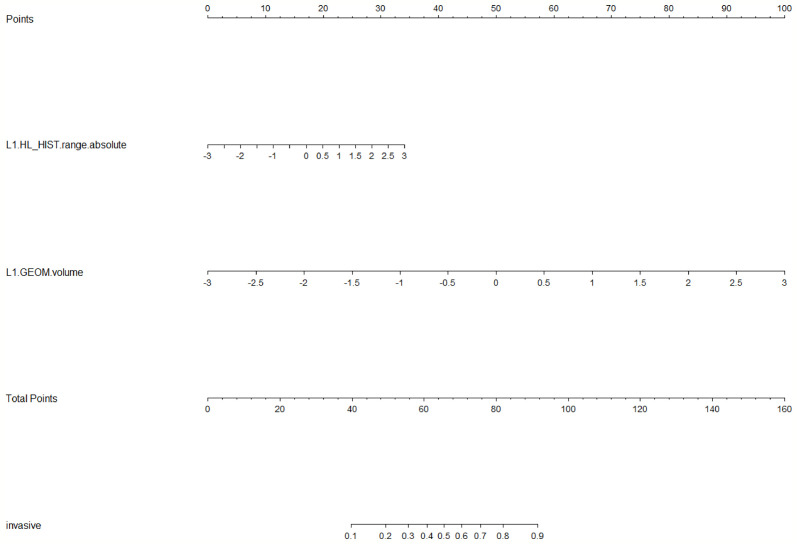
Radiomics nomogram for predicting T stage. Radiomic features, which included HL_HIST range absolute and GEOM volume, corresponded to a point by drawing a vertical line from the location on the HL_HIST range absolute axis or GEOM volume axis up to the point axis. The sum of all points was used to predict the T stage by drawing a vertical line up to the invasive axis, where the closer the line was to the left side of the invasive axis, the lower the T stage.

**Figure 8:**
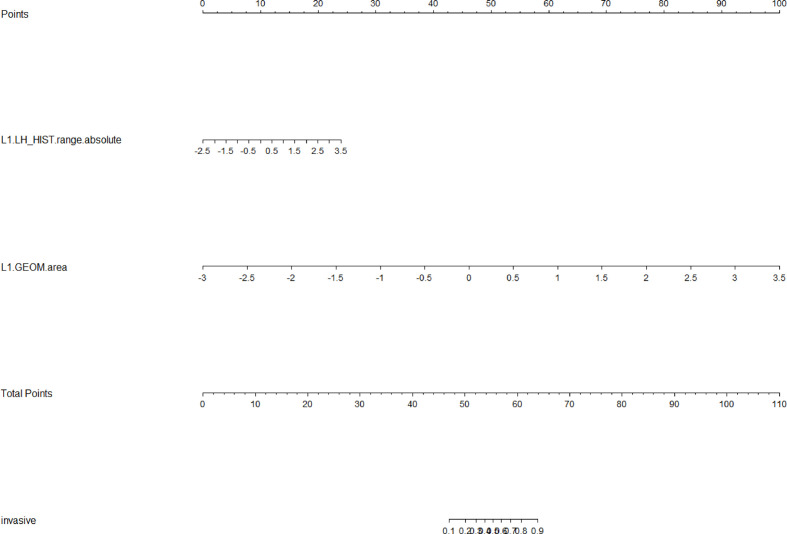
Radiomics nomogram for predicting tumor length. Radiomics features, which included HL_HIST range absolute and GEOM volume, corresponded to a point by drawing a vertical line from the location on the HL_HIST range absolute axis or GEOM volume axis up to the point axis. The sum of all points was used to predict tumor length by drawing a vertical line up to the invasive axis, where the closer the line was to the left side of the invasive axis, the shorter the tumor length.

LASSO analysis was implemented to select radiomics features and build a prediction model. Two radiomics features (L1_HL_HIST_range_absolute, L1_GEOM_volume) were selected out of 251 features, and their regression coefficients were used to construct a radiomics model. The prediction model exhibited favorable differentiating capability for T stage with an AUC of 0.857 (95% confidence interval [CI]: 0.691–1.000, sensitivity: 77.0%, specificity: 87.0%) and excellent predictive ability for tumor length with an AUC of 0.946 (95% CI: 0.862–1.000, sensitivity: 92.0%, specificity: 91.0%; **Figures 5**, **6**). The nomograms for T stage and length are presented in **Figures 7**, **8**.

## Discussion

In the present study, radiomics features were extracted from three-dimensional tumor regions, rather than two-dimensional tumor regions, on the slice with the maximum tumor lesion (8), which provided comprehensive information on esophageal tumors. Subsequently, unsupervised cluster analysis was implemented to categorize patients according to their radiomics features extracted from contrast-enhanced CT images. T stage and tumor length were significantly different between these two clusters, which demonstrated the prediction ability of CT-based radiomics features in patients with ESCC. The supervised LASSO method was then used to select radiomics features and establish radiomics signatures, which confirmed the predictive ability of the radiomics features for T stage and length with high sensitivity and specificity. The combined approach of both the unsupervised and supervised learning methods strengthened the robustness of our results. To the best of our best knowledge, this is the first study to use CT-based radiomics to predict pathological T stage and length in patients with ESCC.

The majority of ESCC patients are diagnosed at advanced stages. For these patients, multimodal strategies, such as neoadjuvant chemoradiotherapy with surgery or primary chemoradiotherapy, can improve clinical outcomes and are considered state-of-the-art by current guidelines (12, 13). However, inaccurate tumor staging may mislead treatment decisions, which hinders positive therapeutic outcomes and consequently patient prognoses. Furthermore, accurate assessment of the T stage is difficult to achieve without surgery and pathological examination. CT and PET-CT play limited roles in determining T stage (14, 15), and EU is the only method that can differentiate the layers of the esophageal wall. However, stenosis and obstruction, which are common symptoms of ESCC patients, limit the application of EU. Moreover, heterogeneity in the experience and subjective proficiency of EU operators are additional obstacles that can bias EU results. Therefore, CT-based radiomics may help overcome the inaccuracy of standard staging methods, which would have a significant impact on ESCC treatment.

Radiomics provides extensive additional information that is contained in images owing to the data-digging process with high-throughput quantitative feature extraction (6). Since it was first introduced, radiomics has rapidly gained the interest of researchers and clinicians. There is not mounting evidence for its predictive ability for tumor biology, stage, treatment response, and prognosis (2, 16–18). Various modalities that enable the acquisition of transverse sectional images, such as CT, magnetic resonance imaging, and PET-CT, can be used for radiomics analysis (19, 20). Contrast-enhanced CT is recommended for routine examination of ESCC and is commonly applied to clinical practice. Therefore, in this study, we chose contrast-enhanced CT to assess radiomics features. In several previous studies, CT-based radiomics in the prediction of treatment response in esophageal malignancies have been explored. Yang et al. demonstrated the predictive performance of complete pathologic response rate after neoadjuvant chemoradiotherapy in a small-scale study, and Hu et al. investigated pre-therapeutic assessment ability for neoadjuvant chemoradiotherapy response in a multicenter study in a larger sample size (21). Furthermore, Liu et al. developed and validated a nomogram based on pretreatment CT radiomics features for predicting complete response to chemoradiotherapy (22). CT-based radiomics has also been applied to tumor stage assessment. Wu et al. reported the potential of CT radiomics in identifying stages I−II and III−IV ESCCs before treatment, with an AUC value of 0.762 (23). However, tumor depth and length were not investigated, although tumor length and invasion depth have previously been shown to be relevant prognostic factors for predicting tumor staging and survival (24). Taken together, these studies highlight the predictive potential of CT radiomics features for ESCC. In this study, we included not only tumor stage but also tumor length and invasion depth. CT radiomics showed favorable prediction ability for T stage, tumor stage could not be discriminated by radiomics in the present study. This may be because of the lack of pathological lymph node information in the analysis and/or the relatively small sample size from a single center. Therefore, we plan to include information on pathologic lymph nodes in our future study.

This study has several limitations. First, it was a relatively small-scale retrospective study. The negative results for OS, PFS, HG, N stage, tumor stage, or VI may have been false-negative results. Large-scale prospective studies are warranted to verify the predictive ability of CT radiomics for ESCC. Second, lymph nodes were not delineated and considered in the analysis, which may have contributed to the inability of radiomics features to predict N stage. Third, OS and PFS were treated as binary variables, and survival prediction was based only on patients’ survival status. Therefore, in future research, the predictive ability of survival span will also be examined.

## Conclusion

Radiomics features and signatures based on contrast-enhanced CT have the potential to predict ESCC T stage and tumor length. Furthermore, because it is based on existing CT imaging data, radiomics has the advantage of being simple to implement, non-invasive, and economical. Combined with conventional staging methods, such as CT, EU, and PET, radiomics may help to achieve more accurate staging and offer optimized cancer treatments. Further prospective large-scale studies are required to verify the predictive role of CT radiomics in patients with ESCC.

## Data Availability Statement

The raw data supporting the conclusions of this article will be made available by the authors, without undue reservation.

## Ethics Statement

The studies involving human participants were reviewed and approved by the institutional review board of the First Affiliated Hospital of Anhui Medical University. Written informed consent for participation was not required for this study in accordance with the national legislation and the institutional requirements.

## Author Contributions

MY conceptualized the idea, designed the work, analyzed the data, and drafted the work. PH extracted the radiomic features from CT images and analyzed the data. ML and RD revised the work and participated in project supervision. YW, SP, WK, and MK collected the data and participated in drafting the work. DD collected the data. FW interpreted the data, revised the work, and participated in project supervision. All authors read and approved the final manuscript. All authors contributed to the article and approved the submitted version.

## Funding

This work was supported by Anhui Medical University Basic Medicine and Clinical Medicine Cooperation Research Promotion Program, Hefei, China [grant number: 2019xkjT025].

## Conflict of Interest

The authors declare that the research was conducted in the absence of any commercial or financial relationships that could be construed as a potential conflict of interest.

## Publisher’s Note

All claims expressed in this article are solely those of the authors and do not necessarily represent those of their affiliated organizations, or those of the publisher, the editors and the reviewers. Any product that may be evaluated in this article, or claim that may be made by its manufacturer, is not guaranteed or endorsed by the publisher.

## Abbreviations

ESCC, esophageal squamous cell carcinoma; CT, computed tomography; AUC, area under the curve; EU, endoscopic ultrasound; PET, positron emission tomography; HG, histological grade; VI, vascular invasion; OS, overall survival; PFS, progression-free survival; VOI, volume of interest; LASSO, the least absolute shrinkage and selection operator; TNM, tumor node metastasis.
